# The Endophytic Fungus *Piriformospora indica* Reprograms Banana to Cold Resistance

**DOI:** 10.3390/ijms22094973

**Published:** 2021-05-07

**Authors:** Dan Li, David Mahoudjro Bodjrenou, Shuting Zhang, Bin Wang, Hong Pan, Kai-Wun Yeh, Zhongxiong Lai, Chunzhen Cheng

**Affiliations:** 1 Institute of Horticultural Biotechnology, Fujian Agriculture and Forestry University, Fuzhou 350002, China; leed6335@126.com (D.L.); davidbodjrenou@ymail.com (D.M.B.); shutingvictory@yeah.net (S.Z.); wb971220@126.com (B.W.); ph734608903@126.com (H.P.); ykwbppp@ntu.edu.tw (K.-W.Y.); 2 College of Horticulture, Shanxi Agricultural University, Taigu 030801, China

**Keywords:** endophytic fungus, banana cold resistance, antioxidant enzymes, soluble sugar, cold-responsive genes

## Abstract

Banana (*Musa* spp.), one of the most important fruits worldwide, is generally cold sensitive. In this study, by using the cold-sensitive banana variety Tianbaojiao (*Musa acuminate*) as the study material, we investigated the effects of *Piriformospora indica* on banana cold resistance. Seedlings with and without fungus colonization were subjected to 4 °C cold treatment. The changes in plant phenotypes, some physiological and biochemical parameters, chlorophyll fluorescence parameters, and the expression of eight cold-responsive genes in banana leaves before and after cold treatment were measured. Results demonstrated that *P. indica* colonization reduced the contents of malondialdehyde (MDA) and hydrogen peroxide (H_2_O_2_) but increased the activities of superoxide dismutase (SOD) and catalase (CAT) and the contents of soluble sugar (SS) and proline. Noteworthily, the CAT activity and SS content in the leaves of *P. indica*-colonized banana were significant (*p* < 0.05). After 24 h cold treatment, the decline in maximum photochemistry efficiency of photosystem II (*F_v_*/*F_m_*), photochemical quenching coefficient (*qP*), efficient quantum yield [Y(II)], and photosynthetic electron transport rate (ETR) in the leaves of *P. indica*-colonized banana was found to be lower than in the non-inoculated controls (*p* < 0.05). Moreover, although the difference was not significant, *P. indica* colonization increased the photochemical conversion efficiency and electron transport rate and alleviated the damage to the photosynthetic reaction center of banana leaves under cold treatment to some extent. Additionally, the expression of the most cold-responsive genes in banana leaves was significantly induced by *P. indica* during cold stress (*p* < 0.05). It was concluded that *P. indica* confers banana with enhanced cold resistance by stimulating antioxidant capacity, SS accumulation, and the expression of cold-responsive genes in leaves. The results obtained from this study are helpful for understanding the *P. indica*-induced cold resistance in banana.

## 1. Introduction

Accumulated reports have shown that the symbiotic interaction between host plants and some of their endophyte microorganisms can confer the host plants with stress tolerance or resistance [1,2]. Among these beneficial microorganisms, *Piriformospora indica* (also named *Serendipita indica*) is one of the most focused on members. As an axenically culturable root-colonizing endophytic fungus, *P. indica* has almost all the beneficial properties of arbuscular mycorrhizal fungi (AMF) and with a broader plant host range [3]. *P. indica* can interact with various plant species, including model plants such as tobacco [4], rice [5], and barley [6], as well as cruciferous plants, such as *Arabidopsis thaliana* [4,7,8,9].

Banana (*Musa* spp.) is one of the most important economic fruits in the world’s import and export trade. However, cultivated banana varieties are usually sensitive to low temperature. Cold damage is harmful to the banana quality, yield, as well as exports in subtropic regions and causes great economic losses. According to reports of the Food and Agriculture Organization (FAO) of the United Nations, four of the seven drops in Ecuador’s banana exports during 1961–2011 were related to climatic changes, including low temperature [10]. The banana-growing areas in China are mostly distributed in the northern boundary of the subtropical region, where chilling or freezing injuries frequently occurs in winter and early spring. In 2015, China’s banana planting area was about 410,000 hectares, with an annual output of more than 12 million tons. In 2016, the planting area reduced to about 380,000 hectares, and the annual output dropped sharply to 8.93 million tons, affected by several factors, including cold snaps [11,12]. Thus, improving cold resistance is essential for the healthy development of the banana industry.

Many studies have demonstrated that symbiotic fungi display positive effects on plant cold tolerance or resistance. Vesicular arbuscular (VA) mycorrhizae and/or AMF fungi have been proved to confer *Zea mays* [13], *Oryza sativa* [14], cucumber [15], and *Elymus nutans* Griseb [16] with resistance to overcome low temperature by enhancing their photosynthetic and antioxidant abilities. *P. indica* colonization can significantly promote the growth of most of its host plants, strengthen the resistance of the plants to various biotic and abiotic stresses, and extensively participate in many plant physiological and biochemical metabolism processes [17,18,19,20,21]. Given that *P. indica* is isolated from the Indian Thar desert, its thermal adaptability is broad. In addition, its potential in helping host plants to resist high and low temperatures has also been reported in recent years [22,23,24,25,26,27]. *P. indica* can promote the seed germination and biomass accumulation of 12 vegetables in high-altitude and low-temperature regions [22,23]. Murphy et al. [24] and Alizadeh et al. [25], respectively, reported the antioxidant capacity-enhancing and growth-promoting effects of *P. indica* on barley and green bean (*Phaseolus vulgaris*) under low-temperature conditions. Moreover, it was reported that *P. indica* can enhance the freezing resistance of *A. thaliana* by upregulating the expression of *CBF*-dependent pathway genes, especially *ICE1* and *CBF1* [26].

The colonization ability of *P. indica* in banana root was first proved by Madaan et al. [28]. In addition, our previous studies have shown that its colonization can improve the growth and rooting of tissue-cultured banana seedlings [29], banana wilt resistance [30], and high-temperature tolerance [27]. However, up to now, there is no report on its influence on banana cold resistance. Numerous studies have credibly revealed that *P. indica*-mediated plant resistance results from the enhancement of antioxidant enzyme activity, the regulation of the expression of stress-responsive genes, and the alleviation of plant cell membrane damage [5,31,32,33,34]. Thus, in our present study, to evaluate the effect of *P. indica* on the cold resistance of banana, we determined several physiological and biochemical indexes (including electrolyte leakage (EL), parameters of chlorophyll fluorescence, activities of superoxide dismutase (SOD), peroxidase (POD), catalase (CAT), polyphenol oxidase (PPO), ascorbate peroxidase (APX), glutathione reductase (GR), and contents of malondialdehyde (MDA), proline, soluble sugar (SS), hydrogen peroxide (H_2_O_2_)) in banana leaves of *P. indica*-colonized and non-colonized control plants. Moreover, to reveal the molecular mechanism of the fungus-induced low temperature in banana, the expression of several reported banana cold-responsive genes, including copper/zinc superoxide dismutase 1C (*CSD1C*) [35], chitinase anti-freeze protein I 1 (*ChiI 1*) [36], transcription factor WHIRLY 1 (*Why 1*) [37], adaptor 1 (*ADA1*) [38,39], and the central stress integrator gene *SNF1-related protein kinase catalytic subunit alpha 10* (*KIN10*) and high expression of osmotically responsive gene 1 (*HOS1*), inducer of CBF expression 1 (*ICE1*) [40], and C-repeat-binding factor 7-1 (*CBF7-1*) [41] were also investigated. The results obtained from this study would be helpful in clarifying the mechanism of the cold resistance enhancement ability of *P. indica* in banana.

## 2. Results

### 2.1. P. indica Colonization Detection Result

**Figure 1:**
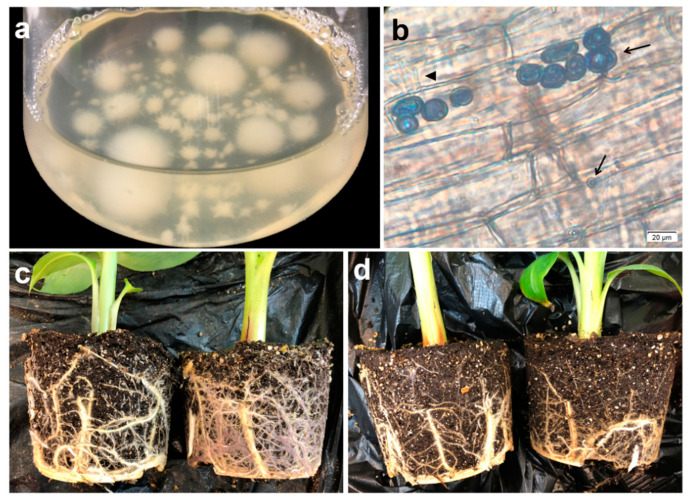
*P. indica* colonization in banana roots. (**a**) *P. indica* inoculation solution and (**b**) typical trypan blue detection result of *P. indica* colonization in banana root. Arrows represent *P. indica* chlamydospores; the triangle represents *P. indica* hyphae. (**c**) Roots of *P. indica*-colonized banana and (**d**) roots of non-inoculated controls.

Trypan blue staining results showed that the chlamydospores and hyphae of *P. indica* colonized successfully in the banana root cortex cells inter- and intracellularly (Figure 1). Moreover, the roots of the *P. indica* group (Pi) were notably stronger than those of the control group (CK) (Figure 1c,d), indicating that *P. indica* colonization promotes the root growth of banana.

### 2.2. The Injury and Influence on Chlorophyll Fluorescence Parameters Caused by Low Temperature in Banana Leaves Could Be Alleviated by P. indica Colonization

**Figure 2:**
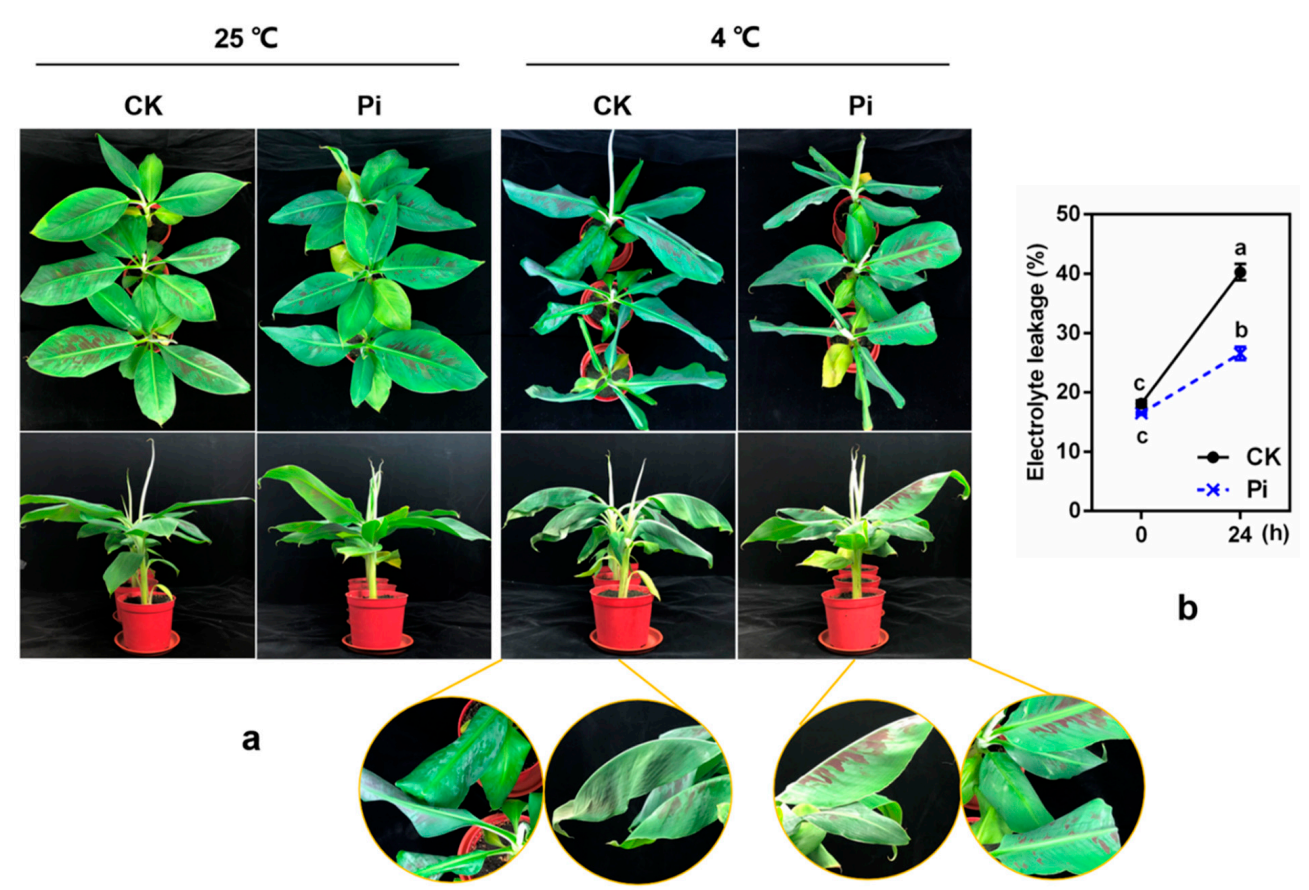
Effect of *P. indica* on the phenotypes (**a**) and relative electrolyte leakage (**b**) of banana before and after 4 °C cold treatment for 24 h. CK: *P. indica* non-colonized control plants; Pi: plants inoculated with *P. indica*. Different letters above the bars indicate a significant difference (*p* < 0.05) from CK (0 h) between CK and Pi groups. Error bars represent SDs (*n* = 3).

After 24 h 4 °C low-temperature treatment, an obvious low-temperature injury difference was found between Pi and CK plants. A significant phenotypic difference was that all CK leaves drooped and curled, and the edges of the top and second leaves displayed severe necrosis, wilting symptoms, and water-stained spots. Meanwhile, Pi plants showed much slighter injury symptoms (Figure 2a). After low-temperature treatment, the relative EL of CK and Pi both decreased significantly (*p* < 0.05), and the EL of Pi was significantly lower than that of CK (*p* < 0.05) (Figure 2b).

**Figure 3:**
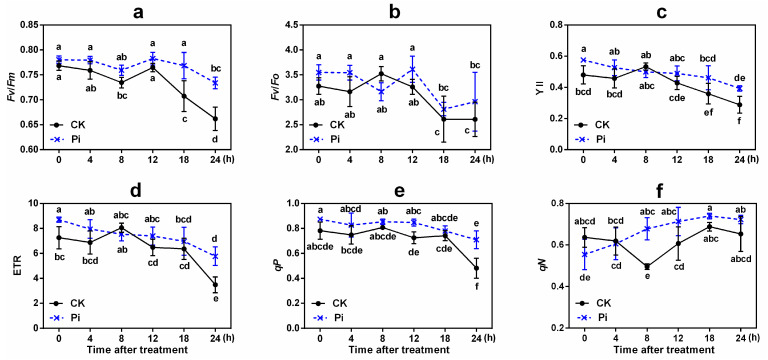
Effects of *P. indica* on chlorophyll fluorescence parameter changes in banana leaves under cold stress treatment. (**a**) *F_v_*/*F_m_*, maximum photochemistry efficiency of PS II; (**b**) *F_v_*/*F_o_*, potential photosynthetic efficiency of PS II; (**c**) Y(II), efficient quantum yield; (**d**) ETR, photosynthetic electron transport rate; (**e**) *qP*, photochemical quenching coefficient; and (**f**) *qN*, non-photochemical quenching coefficient. Different letters above the bars indicate a significant difference (*p* < 0.05) from CK (0 h) between the CK and Pi groups. Error bars represent SDs (*n* = 3).

The change patterns of chlorophyll fluorescence parameters [including *F_v_*/*F_m_* (maximum photochemistry efficiency of photosystem II [PS II]), *F_v_*/*F_o_* (potential photosynthetic efficiency of PS II), Y(II) (efficient quantum yield), ETR (photosynthetic electron transport rate), *qP* and *qN* (photochemical and non-photochemical quenching coefficient)] in Pi and CK also differed (Figure 3). There was a similarity change trend in *F_v_*/*F_m_* in the two groups, but the *F_v_*/*F_m_* values of Pi were higher than those of CK at all the time points, and their difference was significant at 18 and 24 h (*p* < 0.05). The *F_v_*/*F_m_* value decreased significantly in CK leaves after cold treatment for 8 and 18 h and reached the lowest level at 24 h compared with 0 h (*p* < 0.05), while no significant *F_v_*/*F_m_* value difference was found among different time points except at 24 h in Pi. The *F_v_*/*F_o_* values reduced significantly at 18 and 24 h in both groups (*p* < 0.05), and Pi had a higher value than CK except at 8 h (*p* > 0.05). This may be explained by the fact that banana initiates the first cold stress response at 8 h [42,43]. These results indicated that *P. indica* shows a protective effect on the photosynthetic efficiency of banana leaves under cold exposure, thus alleviating the damage by low temperature to the photosynthetic system to a certain extent.

After low-temperature treatment, the Y(II) and ETR values of both CK and Pi also reduced greatly. These values of Pi were all higher than those of CK (*p* < 0.05) except at 8 h (Figure 3). The light protection abilities (*qP*) of Pi after low-temperature treatment were all higher than those of CK. And after 24 h low-temperature treatment, the *qP* value of Pi was found to be significantly higher than that of CK (*p* < 0.05). The value of *qN* increased constantly in Pi, while it decreased in the early stage and increased after 8 h in CK, and the *qN* value of Pi at 8 h after low-temperature treatment was significantly higher than that of CK (*p* < 0.05).

### 2.3. Effects of P. indica on SOD, POD, CAT, and PPO Activities in Banana Leaves under Cold Stress

**Figure 4:**
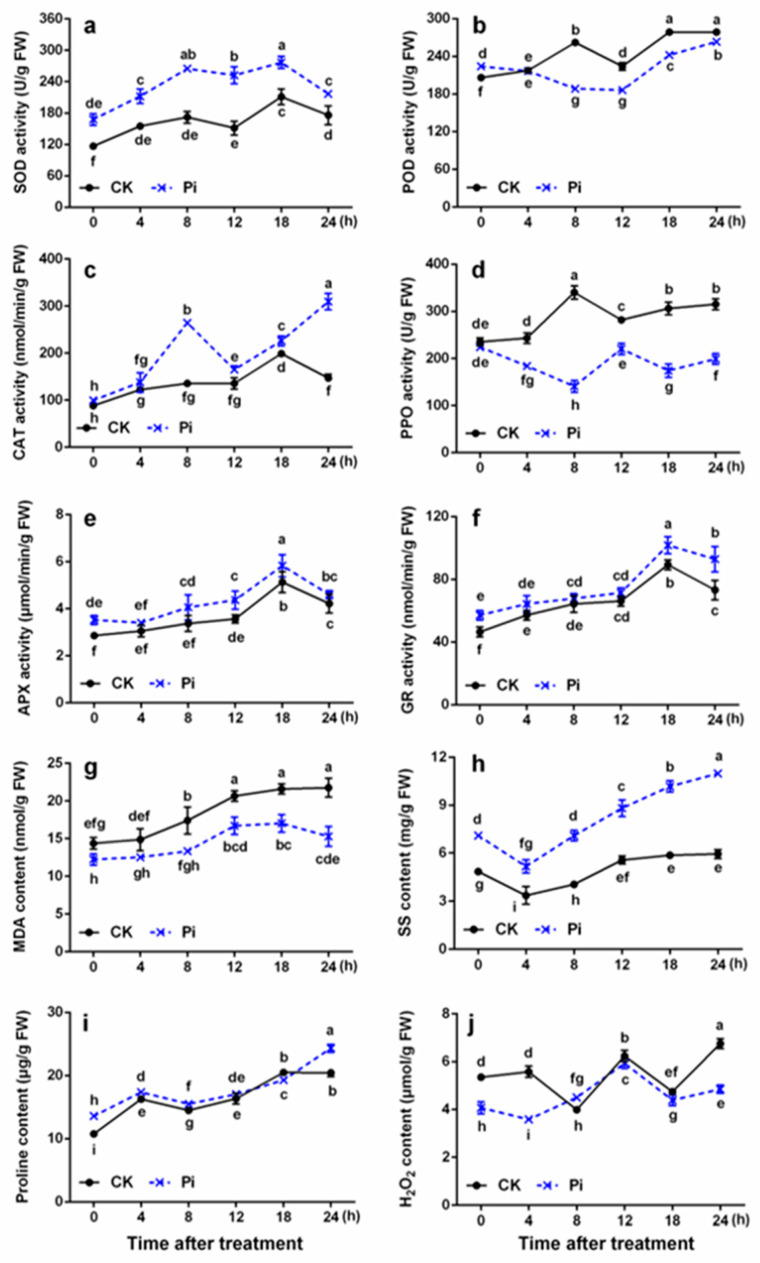
The cold-related physiological and biochemical indexes in banana leaves inoculated or non-inoculated with *P. indica* under cold stress. (**a**) Superoxide dismutase activity; (**b**) peroxidase activity; (**c**) catalase activity; (**d**) polyphenol oxidase activity; (**e**) ascorbate peroxidase activity; (**f**) glutathione reductase activity; (**g**) malondialdehyde content; (**h**) soluble sugar content; (**i**) proline content; and (**j**) hydrogen peroxide content. FW, fresh weight; CK, control, plants non-inoculated with *P. indica*; Pi, plants inoculated with *P. indica*. Different letters above the bars indicate a significant difference (*p* < 0.05) from CK (0 h) between CK and Pi groups. Error bars represent SDs (*n* = 3).

The SOD activity in Pi was significantly higher than that in CK before and after low-temperature treatment (*p* < 0.05) (Figure 4a). Pi plants had higher POD activity than CK plants at 0 h, which showed a trend of falling first and then rising after 8 h. However, the POD activity of CK plants increased with the extension of cold stress time before 8 h, decreased at 12 h, and increased again after 18 h (Figure 4b). This phenomenon might be closely related to the reactive oxygen species (ROS) content variation in banana leaves. Notably, the CAT activity of Pi increased by 211.4% after 24 h low-temperature treatment compared with the 0 h control, while that of CK increased by only about 66.7% (Figure 4c). The PPO activity was apparently inhibited in Pi plants; it reduced by 11.3% after being exposed to low temperature for 24 h compared with 0 h (*p* < 0.05), whereas it increased obviously by about 34.0% in CK plants (*p* < 0.05) (Figure 4d).

### 2.4. Effects of P. indica on APX and GR Activities in Banana Leaves under Cold Stress

The APX and GR activities in CK and Pi showed similar trends during cold stress, increased with the extension of low-temperature treatment time, and peaked at 18 h (*p* < 0.05). Pi plants had higher APX activity than CK plants at all the time points (Figure 4e). Except at 4 and 24 h, the APX activity of Pi was significantly higher than that of CK (*p* < 0.05). In addition, the GR activity in Pi and CK leaves, respectively, increased by about 62.8% and 57.5% after cold stress of 24 h compared with 0 h (Figure 4f).

### 2.5. Effects of P. indica on MDA, SS, Proline, and H_2_O_2_ Contents of Banana Leaves under Cold Stress

The MDA content of leaves increased by about 51.5% after cold treatment for 24 h in CK but only by 25.4% in Pi (Figure 4g). The H_2_O_2_ content of CK and Pi increased by about 26.2% and 19.2%, respectively, after cold stress for 24 h compared with 0 h. Moreover, the H_2_O_2_ content of Pi was only about 76% of that of CK and was significantly lower than that of CK at 0 h *(p* < 0.05) (Figure 4j). The SS content of Pi plants was about 1.46 times that of CK at 0 h and increased by 54.9% after 24 h compared with 0 h (Figure 4h). The proline content of Pi was significantly *(p* < 0.05) higher than that of CK except at 12 and 18 h (Figure 4i).

### 2.6. Effects of P. indica on the Expression of Cold-Responsive Genes in Banana Leaves under Cold Stress

**Figure 5:**
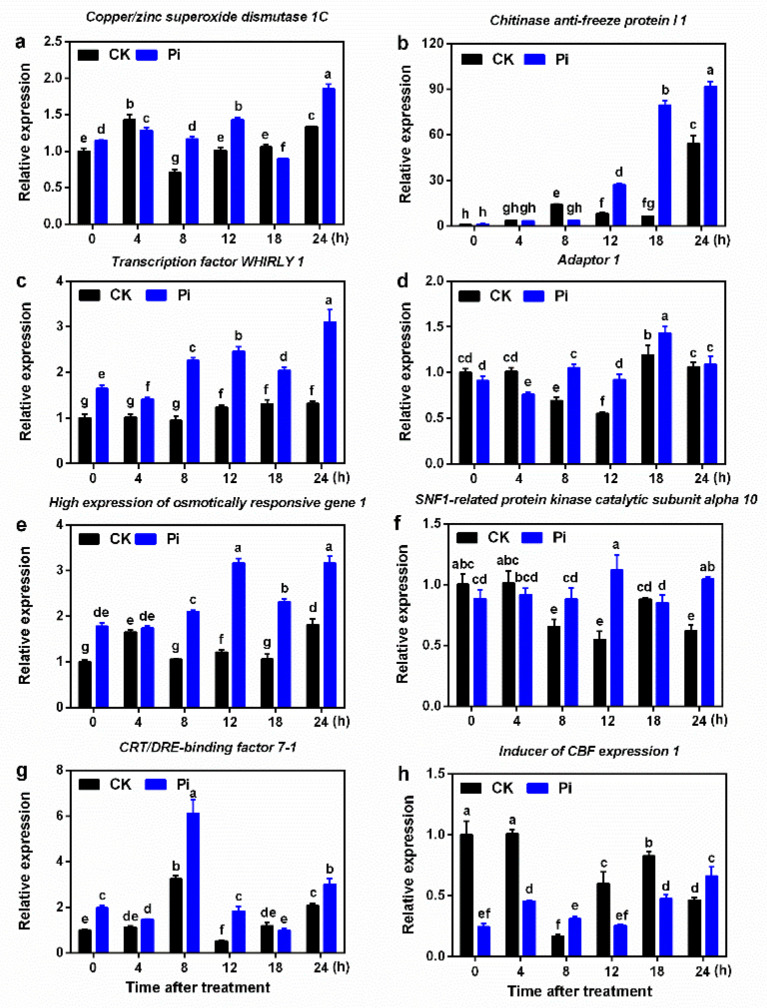
The relative gene expression levels of (**a**) copper/zinc superoxide dismutase 1C (*CSD1C*), (**b**) chitinase anti-freeze protein I 1 *(ChiI 1*), (**c**) transcription factor WHIRLY 1 (*Why 1*), (**d**) *ADA1*, adaptor 1, (**e**) high expression of osmotically responsive gene 1 (*HOS1*), (**f**) SNF1-related protein kinase catalytic subunit alpha 10 (*KIN10*), (**g**) CRT/DRE-binding factor 7-1 (*CBF7-1*) and (**h**) inducer of CBF expression 1 (*ICE1*) from *P. indica*-colonized and non-colonized banana were analyzed and compared across the time course of cold stress. Values represent means ± standard deviation of three replicates. Different lowercase letters above the bars indicate statistically significant differences at the 0.05 level from CK (0 h) between the CK and Pi groups by Duncan’s multiple-range test. Error bars represent SDs (*n* = 3).

Compared with CK plants at 0 h, the expression of *CSD1C*, *Why 1*, *HOS1*, and *CBF7-1* genes was significantly induced by *P. indica* colonization *(p* < 0.05, Figure 5). Interestingly, the relative expression of *CSD1C, ADA1*, and *KIN10* genes was remarkably downregulated in CK plants after cold stress for 8 h *(p* < 0.05), while it showed an upregulation trend affected by *P. indica* and their expression in Pi was significantly higher than that in CK *(p* < 0.05). The *ChiI 1* gene showed a rise-fall-rise expression pattern in CK but a rising pattern in Pi during cold stress (Figure 5b). Its expression level at 8 and 24 h in CK was 14.06 and 54.34 times that of the 0 h control, respectively. Notably, its expression at 12, 18, and 24 h in Pi was 20.33, 59.82, and 69.03 times that of the 0 h control, respectively. ICE1 is recognized as a negative regulator of plant cold resistance. In this study, the expression of *ICE1* in Pi was lower than that in CK at 0, 4, 12, and 18 h but higher than that in CK at 8 and 24 h. After 12 h low-temperature treatment, the expression of all these genes except *ICE1* was noticeably upregulated in Pi plants (Figure 5h). At 24 h, the expression levels of all these genes in Pi were higher than in CK.

## 3. Discussion

In the present study, we first investigated the influence of *P. indica* colonization on banana cold resistance. After 24 h 4 °C treatment, the *P. indica*-colonized banana plants showed much milder cold injury symptoms and lower relative EL compared with the non-colonized plants (Figure 2). Chlorophyll fluorescence reflects the state of the PS II reaction center [44]. Low temperature can cause damage to the PS II reaction center, which reduces the photosynthetic activity and electron transfer rate of plant leaves, enhances photoinhibition, and decreases *F_v_*/*F_m_* and *F_v_*/*F_o_* [45,46]. The colonization of *P. indica* in *A. thaliana* under salt stress leads to the increase in effective transfer of electron flow in PS II, which improves the level of photochemical and non-photochemical quenching and alleviates the damage by salt stress to plants [47,48]. The *P. indica*-non-colonized *Arabidopsis* seedlings showed performed a much faster decrease in *F_v_*/*F_m_* and *qP* values after being exposed to freezing treatment than the *P. indica*-colonized ones [26]. In this study, although no significant difference was found, the decline in *F_v_*/*F_m_*, *F_v_*/*F_o_*, Y(II), and ETR of *P. indica*-colonized banana leaves was lower than that in non-colonized controls under low temperature to some extent (Figure 3). *qP* is positively correlated with plant photosynthetic activity, and low temperature often results in a decrease in *qP* [26,49]. *qN* indicates the ability of light protection, that is, the ability of plants to dissipate excess light energy through thermal dissipation [49]. The *qP* and *qN* values of *P. indica*-colonized banana leaves before and after low-temperature treatment were almost higher than those of the non-colonized controls (significant *qP* difference was found between Pi and CK at 12 and 24 h, and significant *qN* difference was found at 8 h (*p* < 0.05). Thus, it is suspected that *P. indica*-colonized banana may have a better photosynthetic capacity than non-inoculated plants.

The balance between the generation and clearance of ROS, which acts as a signal transduction molecule that regulates plant–microbial interactions, is essential for the adaptive defense responses of plants [50]. Cold-sensitive plants usually have weak ROS-scavenging ability when encountering low-temperature stress. *P. indica* can help plants regulate and control the antioxidant system when plants are exposed to stresses. Garg et al. [51] reported that the antioxidant enzyme activity of mycorrhizal plants is significantly higher than that of non-mycorrhizal plants to alleviate the damages caused by stress. *P. indica* can promote the growth of barley and green beans under low-temperature stress [24,25], which indicates that *P. indica* has the potential to enhance plant cold resistance. In this study, higher SOD, CAT, APX, and GR activities were found in *P. indica*-colonized banana leaves both before and after low-temperature treatment (*p* < 0.05; Figure 4), indicating that *P. indica* colonization increases the activities of these enzymes in banana leaves and enhances the ROS-scavenging ability of banana under cold stress. Generally, low-temperature stress increases the lipid peroxidation of the cell membrane and induces the accumulation of MDA and H_2_O_2_ [52]. The *P. indica* colonization reduced the MDA and H_2_O_2_ contents of banana leaves (*p* < 0.05) and alleviated the cell damage caused by membrane lipid peroxidation to the leaves at low temperature. Our results were consistent with those obtained by Han et al. [53], in whose study the AMF was reported to reduce the MDA content of cucumber seedlings under cold stress. *P. indica* inoculation remarkably inhibited PPO activity during the low temperature (*p* < 0.05), and there was no significant difference in basic PPO activity between Pi and CK at 0 h (*p* > 0.05). In addition, *P. indica* colonization actually affected the phenotype of banana caused by cold to a certain extent, and the necrosis, wilting, and water-stained spot symptoms of Pi were greatly milder than those of CK after cold stress. The lower PPO activity in the leaves of *P. indica*-colonized banana may well explain this phenomenon. APX is one of the important antioxidant enzymes for ROS scavenging in plants, as well as a key enzyme in ascorbic acid metabolism, which catalyzes the oxidation of AsA by H_2_O_2_ [18]. H_2_O_2_ is a vital reactive oxygen molecule, CAT is a major H_2_O_2_-scavenging enzyme, and APX can scavenge H_2_O_2_ with ASA as an electron donor. Alizadeh et al. [25] studied the effects of *P. indica* on green bean cold resistance and found that the CAT activity of *P. indica*-inoculated plants was 2.16 times higher compared to non-inoculated ones after 3 days under cold stress. Similarly, the CAT activity in Pi noticeably increased by 211.4% after 24 h of cold stress compared with 0 h (*p* < 0.05; Figure 4). Besides, the H_2_O_2_ content showed a negative correlation with CAT activity. This indicated that *P. indica* increases CAT activity, participated in the elimination of excess free radicals, accelerates the scavenging efficiency of H_2_O_2_, and mitigates the damage of the cell membrane structure caused by cold stress. At 0 h, Pi had a higher APX activity than CK at 18 h after cold stress (*p* < 0.05). Therefore, it can be inferred that higher APX activity may be involved in the defense mechanism of *P. indica* protecting banana from cold stress. Liu et al. [54] reported that the colonization of *Glomus mosseae* increases the activities of SOD and APX and the contents of SS and proline in blueberry leaves under cold stress and reduces MDA and H_2_O_2_ contents to enhance cold resistance. GR is one of the key enzymes in the glutathione redox cycle and participates in the AsA-GSH cycle pathway. It catalyzes NADPH to reduce GSSG to regenerate GSH and plays a vital role in the ROS-scavenging reaction to oxidative stress [18,55]. In our present study, fungal colonization significantly increased the GR activity in banana leaves both before and after cold stress (*p* < 0.05). Chu et al. [16] reported that AMF-inoculated plants have a higher SS content than non-inoculated ones under cold exposure. Sharma et al. [56] reported that *P. indica* inoculation increases the reducing sugar content of *Aloe vera* under stress conditions. In this study, we found that *P. indica* can significantly promote the accumulation of SS in banana leaves (*p* < 0.05). Thus, it was suspected that SS biosynthesis contributes to the *P. indica*-induced banana cold resistance.

CSD, disproportioning superoxide anions to produce O_2_ and H_2_O_2_, is vital in scavenging active oxygen in plants and maintaining the balance of active oxygen [57]. *CSD1C* is a cytoplasmic *Cu*/*ZnSOD* gene that reduces plant cell oxidative damage and is related to the cold stress response in banana [35]. The SOD activity and *SOD* expression in Pi before and after cold stress were both significantly higher than in non-colonized plants (*p* < 0.05; Figure 4 and Figure 5). It is suggested that *P. indica* may induce the expression of the *SOD* gene to regulate the cold resistance in banana. Zhang [36] cloned the chitinase anti-freeze protein gene *ChiI 1*, related to glycosidase and hydrolase, from cold-tolerant Sanming wild banana and found that cold stress induces *ChiI 1* expression after 8 h of low-temperature treatment. In the present study, we found that *ChiI 1* was upregulated by cold stress and by *P. indica* colonization in the cold-sensitive cultivar Tianbaojiao banana (*p* < 0.05; Figure 5), indicating that this gene is both low-temperature and *P. indica* responsive. Yang [37] showed that *Why 1* is sensitive to different temperatures and is involved in the regulation of cold resistance pathways in banana. Liu et al. [40,41] cloned *HOS1 CBF7-1*, and *ICE1* genes of the *CBF*-dependent cold resistance pathway and the central stress integrator gene *KIN10* from wild banana. The *ADA1* gene was one of the cold-resistance-related overlapping genes cloned from Sanming wild banana [38,39]. They may all participate in the regulation of the cold resistance pathway in banana. Interestingly, *Why 1* and *HOS1* genes were affected strongly by *P. indica*, and they had a significantly higher expression level than in non-inoculated plants during the entire cold stress process (*p* < 0.05). In addition, *ADA1* and *KIN10* genes were also found to be upregulated by *P. indica*. It is suspected that these genes may play considerable roles in the *P. indica*-induced cold resistance of banana. Wu et al. [42] reported that grape seedlings initiate cold responses twice under cold stress, which have a higher expression at 8 and 48 h. Chen [43] also reported cold responses to low temperature twice in Sanming wild banana. In this study, the expression of almost all genes in Pi at 8 and 24 h after low-temperature treatment was significantly higher than in CK (*p* < 0.05), indicating that *P. indica* enhances the twice cold response of the cold-sensitive Tianbaojiao banana. It was concluded that *P. indica* colonization reprograms banana to cold resistance by regulating not only *CBF*-dependent pathway genes but also some other cold acclimation pathway genes [26,40].

**Figure 6:**
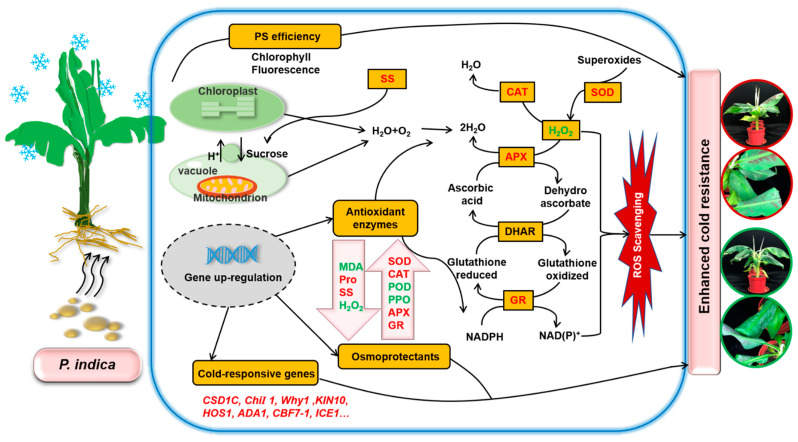
Schematic representation of *Piriformospora indica* symbiotic association-mediated banana cold stress tolerance. PS efficiency, antioxidant enzymes, osmoprotectants, and cold-responsive genes played an essential beneficial role in *P. indica*-induced cold resistance in banana. The parameters and gene names in red indicate a higher level and upregulation, while the green color indicates a lower level and downregulation. This figure is drawn according to Kumar et al. [58], Gill et al. [59], and Nath et al. [50].

Based on the results obtained in this study, we put forward a draft model classifying how *P. indica* colonization alleviates the damage caused by low temperature in banana (Figure 6). Under low-temperature stress, the fungus helped host plants accelerate ROS scavenging, enhance PS efficiency, and elevate plant cell protective enzyme activities and osmoprotectant contents, especially soluble sugar, to alleviate the peroxidative damage to leaf cells caused by cold stress. Moreover, *P. indica* can confer cold resistance to banana by stimulating the expression of cold-responsive genes.

## 4. Materials and Methods

### 4.1. Plant Material and Fungal Inoculation

Rooted in vitro plantlets of Tianbaojiao banana (*Musa acuminata* cv. Tianbaojiao) were transplanted to plastic pots containing nutrient soil. After growing for 2 months in a growth chamber (GXZ-280C, Ningbo Jiangnan Instrument Factory, Ningbo, China) at 25 °C, a photoperiod of 16/8 h (day/night, 1500 ± 200 lx), and 60–80% relative humidity, seedlings at the five- to six-leaf stage were used for *P. indica* (strain DSM11827) inoculation. The *P. indica* inoculation solution (adjusted to a concentration of 2 × 10^5^ chlamydospores/mL) was obtained, and banana roots were inoculated with a content of 100 mL/kg soil according to the method described by Cheng et al. [30]. Plants treated with euvolemia-diluted potato dextrose broth (PDB) solution were used as controls.

### 4.2. P. indica Colonization Detection and Cold Treatment

Two weeks after *P. indica* inoculation, banana roots were randomly collected, washed thoroughly with running tap water, and then cut into 1 cm segments for trypan blue staining [29,60]. Uniform and well-growing banana seedlings inoculated (Pi group) and non-inoculated (CK group) with *P. indica* were exposed to 4 °C cold treatment in growth chamber at 25 °C, a photoperiod of 16/8 h (day/night, 1500 ± 200 lx), and 60–80% relative humidity. In total, 18 plants were used for each group. After cold treatment, the first fully expanded top leaves were harvested at each time point (4 °C for 0, 4, 8, 12, 18, and 24 h), frozen in liquid N_2_ immediately, and finally stored at −80 °C for further use.

### 4.3. Relative Electrolyte Leakage Measurement

Briefly, 0.1 g of leaves (round pieces of 0.5 cm diameter) were collected in tubes containing 10 mL of ultrapure water, evacuated for 20 min using a vacuum pump (SHZ-Ⅲ, Linhai Tanshi Vacuum Equipment Co., Ltd., Linhai, China), and incubated at 25 °C for 30 min. Then, the relative EL was measured and calculated according to the method described by Yang et al. [61].

### 4.4. Chlorophyll Fluorescence Measurement and Leaf Sample Harvesting

Chlorophyll fluorescence parameters, including the maximum photochemistry efficiency of PS II (*F_v_*/*F_m_*), potential photosynthetic efficiency of PS II (*F_v_*/*F_o_*), photochemical and non-photochemical quenching coefficient (*qP* and *qN*), efficient quantum yield (Y(II)), and photosynthetic electron transport rate (ETR), of the second top leaf of three plants from each group were measured at 0, 4, 8, 12, 18, and 24 h after cold treatment with a MAX-IMAGING-PAM instrument (Heinz Walz GmbH, Effeltrich, Germany) [30].

### 4.5. Assay of Antioxidant Enzyme Activity

Briefly, leaf samples were quickly ground into a powder in mortars with liquid N_2_. For each sample and each biological parameter, 0.1 g of leaf powder was homogenized with 1 mL of extraction solution on ice, transferred to cooling tubes, and centrifuged at 4 °C to collect the supernatant for detecting the activities of SOD, POD, CAT, GR, and APX according to the method described by Cheng et al. [30]. For PPO activity, the sample supernatant was collected and used as a crude enzyme (the control tube contained distilled water), added to the reagent solution in a test tube, and put in a 25 °C water bath for 10 min and then at 95 °C for 5 min. It was mixed well and centrifuged at 25 °C (10,000× *g*, 10 min) to obtain the supernatant. One unit (U) of PPO activity was defined as the amount of enzyme causing a change of 0.01 in the absorbance at 525 nm per minute per milliliter of reaction system of per gram of sample.

### 4.6. Measurement of MDA, H_2_O_2_, Proline, and Soluble Sugar Contents

The membrane-permeable related parameters were measured using assay kits (Suzhou Comin Biotechnology Co, Ltd., Suzhou, China) with an ultraviolet–visible spectrophotometer (PerkinElmer, New York, NY, USA) according to the manufacturer’s instructions. For MDA, proline, SS, and H_2_O_2_ content detection, a powdery sample (0.1 g) was homogenized with 1 mL of extraction solution (H_2_O_2_ and SS used acetone solution and distilled water, respectively) on ice, the homogenate mixture was transferred to cooling tubes, and then it was centrifuged at 4 °C (8000× *g*, 10 min) to collect supernatant mixtures. For proline and SS, mixtures were placed in a 95 °C water bath with shaking for extraction for 10 min and centrifuged at 25 °C (10,000 g, 10 min) to collect the supernatant after cooling.

MDA condenses with thiobarbituric acid to produce a red product, which has a maximum absorption peak at 532 nm. The content of lipid peroxide in the sample can be estimated by colorimetry. At the same time, absorbance at 600 nm was determined, and the content of MDA was calculated by the difference between the absorbance at 532 nm and 600 nm. Proline was extracted with sulfosalicylic acid, and after heating, proline reacted with acidic ninhydrin solution to produce a red color. Toluene was added to the extract, and absorbance was scanned at 520 nm. The SS content was determined by anthrone colorimetry according to Sun et al. [62] with a minor modification. The sample supernatant was made up to a final volume of 10 mL with distilled water. Then, 200 μL of the sample and 200 μL of distilled water (400 μL of distilled water as the control) were added into tubes containing 100 μL of the test solution. Next, 1 mL of concentrated sulfuric acid was added to the mixture. The mixture was kept in a 95 °C water bath for 10 min. After cooling, the SS content was measured and calculated under absorbance at 620 nm. H_2_O_2_ forms a yellow titanium peroxide complex with titanium sulfate and has a characteristic absorption at 415 nm.

### 4.7. Analysis of Gene Expression by qRT-PCR

Total RNA was extracted from banana leaves using the RNAprep Pure Plant Kit (TIANGEN, Beijing, China). After RNA quality and quantity examination using 1% agarose gel electrophoresis and NanoDrop 2000 Spectrophotometer (Thermo Fisher Scientific, Waltham, MA, USA), high-quality RNA was reverse-transcribed into cDNA using the PrimerScriptTM RT Reagent Kit (Perfect Real Time; Takara, Shiga, Japan).

Quantitative real-time PCR (qRT-PCR) for gene expression analysis was performed, as described by Lin and Lai [63], using a LightCycler480 Real-time PCR detection instrument (Roche, Basel, Switzerland) with TB Green™ Premix EX Taq™ II (Tli RNaseH Plus; Takara, Shiga, Japan). The specificity of each primer pair was tested and verified by analyzing the melting curve. The *CAC* gene was used as a reference gene [64]. Relative expression levels were determined using the comparative 2^−∆∆Ct^ method. All treatments were analyzed with biological triplicates. The primers used are listed in Table 1.

### 4.8. Statistics Analysis

Results for physiological and biochemical indexes and relative gene expression levels are presented as the mean ± standard deviation (SD) of three biological replicates. All the data analyses were performed using IBM^®^ SPSS^®^ Statistics version 22.0 (IBM Corporation, Armonk, NY, USA) and analyzed by Duncan’s multiple-range test at a 5% significance level. Figures were drawn using GraphPad Prism v6.01 (GraphPad Software, Inc., San Diego, CA, USA).

## Figures and Tables

**Table 1:** Oligonucleotide primers used for qRT-PCR analysis.

Gene	Accession No.	Primer Sequences (5′→3′)	Tm (°C)	Reference
*CSD1C*	KM017514	F: GCCGATCCAGATGATCTTG	57	[35]
		R: ACCTCACCATCCAACCAATAG		
*ChiI 1*	FJ858155	F: CCTTGTTGTTGGTCATCTTTACC	58	[36]
		R: ATTGGCTCTGGCATCCTTGG		
*Why1*	KJ637331	F: GCTTCCTCTCATGCAGCTCT	60	[37]
		R: TGGTGACACTGACAAGGCAG		
*ADA1*	MG451820	F: CTGTGGAAGATGGGGAAGAG	59	[38,39]
		R: CTCGTATCTGGCAGTTGAGAAG		
*HOS1*	JX678611	F: GATCGGCATTGGAAGAGAC	56	[40]
		R: GAGAACTTGAGCTATCTTCGG		
*KIN10*	KC127685	F: GAGATTCGAGAACATCCATGG	58	[40]
		R: ACTCATAGCACGGAACCTATTG		
*CBF7-1*	KC157575.1	F: GAGTAAGCATCCGGTGTACC	57	[41]
		R: CAGATCCGCGACTTCTTG		
*ICE1*	KC157569	F: GAACTCCGCAAGCCAATTC	58	[40]
		R: CTTCCTCCAATGCAGCCA		
*CAC*	HQ853240	F: AACTCCTATGTTGCTCGCTTATG	57	[64]
		R: GGCTACTACTTCGGTTCTTTCAC		

F: forward primer; R: reverse primer.

## Data Availability

Not applicable.

## Abbreviations

SOD	superoxide dismutase
POD	peroxidase
CAT	catalase
PPO	polyphenol oxidase
MDA	malondialdehyde
SS	soluble sugar
H_2_O_2_	hydrogen peroxide
APX	ascorbate peroxidase
GR	glutathione reductase
AsA	ascorbic acid
NADPH	nicotinamide adenine dinucleotide phosphate
GSSG	glutathione oxidized
GSH	reduced glutathione
PS	photosynthetic system
*F_v_/F_m_*	ratio of variable to maximal fluorescence- maximum photochemistry efficiency of PS II
*F_v_/F_o_*	potential photosynthetic efficiency of PS II
*qP*	photochemical quenching coefficient
*qN*	non-photochemical quenching coefficient
Y(II)	efficient quantum yield
ETR	photosynthetic electron transport rate
*CSD1C*	copper/zinc superoxide dismutase 1C
*ChiⅠ 1*	chitinase anti-freeze protein Ⅰ 1
*Why 1*	transcription factor WHIRLY 1
*ADA1*	adaptor 1
*HOS1*	high expression of osmotically responsive gene 1
*ICE1*	inducer of CBF expression 1
*KIN10*	SNF1-related protein kinase catalytic subunit alpha 10
*CBF7-1*	CRT (C-repeat/dehydration response element)/DRE (dehydration responsive element)-binding factor 7-1

## References

[1] Waller F., Achatz B., Baltruschat H., Fodor J., Becker K., Fischer M., Heier T., Huckelhoven R., Neumann C., von Wettstein D. (2005). The endophytic fungus *Piriformospora indica* reprograms barley to salt-stress tolerance, disease resistance, and higher yield. Proc. Natl. Acad. Sci. USA.

[2] Kumar M., Yadav V., Tuteja N., Johri A.K. (2009). Antioxidant enzyme activities in maize plants colonized with *Piriformospora indica*. Microbiology.

[3] Varma A., Verma S., Sudha, Sahay N., Bütehorn B., Franken P. (1999). Piriformospora indica, a cultivable plant-growth-promoting root endophyte. Appl. Environ. Microbiol..

[4] Sherameti I., Shahollari B., Venus Y., Altschmied L., Varma A., Oelmüller R. (2005). The endophytic fungus *Piriformospora indica* stimulates the expression of nitrate reductase and the starch-degrading enzyme glucan-water dikinase in tobacco and *Arabidopsis* roots through a homeodomain transcription factor that binds to a conserved motif in their promoters. J. Biol. Chem..

[5] Bagheri A.A., Saadatmand S., Niknam V., Nejadsatari T., Babaeizad V. (2014). Effects of *Piriformospora indica* on biochemical parameters of *Oryza sativa* under salt stress. Int. J. Biosci..

[6] Baltruschat H., Fodor J., Harrach B.D., Niemczyk E., Barna B., Gullner G., Janeczko A., Kogel K.H., Schäfer P., Schwarczinger I. (2008). Salt tolerance of barley induced by the root endophyte *Piriformospora indica* is associated with a strong increase in antioxidants. New Phytol..

[7] Peškan-Berghöfer T., Shahollari B., Giong P.H., Hehl S., Markert C., Blanke V., Kost G., Varma A., Oelmüller R. (2004). Association of *Piriformospora indica* with *Arabidopsis thaliana* roots represents a novel system to study beneficial plant-microbe interactions and involves early plant protein modifications in the endoplasmic reticulum and at the plasma membrane. Physiol. Plant..

[8] Kumari R., Kishan H., Bhoon Y.K., Varma A. (2003). Colonization of cruciferous plants by *Piriformospora indica*. Curr. Sci..

[9] Qiang X., Weiss M., Kogel K.H., Schäfer P. (2012). *Piriformospora indica*—A mutualistic basidiomycete with an exceptionally large plant host range. Mol. Plant Pathol..

[10] Elbehri A., Calberto G., Staver C., Hospido A., Skully D., Siles P., Arguello J., Sotomayaor I., Bustamante A., Elbehri A. (2016). Ecuador’s Banana Sector under Climate Change: An Economic and Biophysical Assessment to Promote a Sustainable and Climate-Compatible Strategy.

[11] Zhang H. (2016). Development report and situation forecast of banana industry in 2015. World Trop. Agric. Inf..

[12] Wang F., Guo J., Ke Y., Li C. (2017). China banana industry development report 2016 and development trend 2017. China Trop. Agric..

[13] Charest C., Dalpé Y., Brown A. (1993). The effect of vesicular-arbuscular mycorrhizae and chilling on two hybrids of *Zea mays* L. Mycorrhiza.

[14] Liu Z.L., Li Y.J., Hou H.Y., Zhu X.C., Rai V., He X.Y., Tian C.J. (2013). Differences in the arbuscular mycorrhizal fungi-improved rice resistance to low temperature at two N levels: Aspects of N and C metabolism on the plant side. Plant Physiol. Biochem..

[15] Chen S., Jin W., Liu A., Zhang S., Liu D., Wang F., Lin X., He C. (2013). Arbuscular mycorrhizal fungi (AMF) increase growth and secondary metabolism in cucumber subjected to low temperature stress. Sci. Hortic..

[16] Chu X.T., Fu J.J., Sun Y.F., Xu Y.M., Miao Y.J., Xu Y.F., Hu T.M. (2016). Effect of arbuscular mycorrhizal fungi inoculation on cold stress-induced oxidative damage in leaves of *Elymus nutans* Griseb. S. Afr. J. Bot..

[17] Camehl I., Sherameti I., Venus Y., Bethke G., Varma A., Lee J., Oelmüller R. (2010). Ethylene signalling and ethylene-targeted transcription factors are required to balance beneficial and nonbeneficial traits in the symbiosis between the endophytic fungus *Piriformospora indica* and *Arabidopsis thaliana*. New Phytol..

[18] Nanda R., Agrawal V. (2018). *Piriformospora indica*, an excellent system for heavy metal sequestration and amelioration of oxidative stress and DNA damage in *Cassia angustifolia* Vahl under copper stress. Ecotoxicol. Environ. Saf..

[19] Khatabi B., Molitor A., Lindermayr C., Pfiffi S., Durner J., von Wettstein D., Kogel K.-H., Schäfer P. (2012). Ethylene supports colonization of plant roots by the mutualistic fungus *Piriformospora indica*. PLoS ONE.

[20] Sun C., Shao Y., Vahabi K., Lu J., Bhattacharya S., Dong S., Yeh K.W., Sherameti I., Lou B., Baldwin I.T. (2014). The beneficial fungus *Piriformospora indica* protects *Arabidopsis* from *Verticillium dahliae* infection by downregulation plant defense responses. BMC Plant Biol..

[21] Sun C., Shao Y., Vahabi K. (2014). *Piriformospora indica* is an efficient biocontrol agent against Verticillium dahliae wilt: Role of phytohormone signaling in the three partite interaction. J. Endocytobiosis Cell Res..

[22] Varma A., Bakshi M., Lou B., Hartmann A., Oelmueller R. (2012). *Piriformospora indica*: A novel plant growth-promoting mycorrhizal fungus. Agric. Res..

[23] Varma A., Sowjanya Sree K., Arora M., Bajaj R., Prasad R., Kharkwal A.C. (2014). Functions of novel symbiotic fungus—*Piriformospora indica*. Proc. Indian Natl. Sci. Acad..

[24] Murphy B.R., Doohan F.M., Hodkinson T.R. (2014). Yield increase induced by the fungal root endophyte *Piriformospora indica* in barley grown at low temperature is nutrient limited. Symbiosis.

[25] Alizadeh F.M., Pirdashti H., Yaghoubian Y., Babaeizad V. (2016). Effect of paclobutrazol and *Piriformospora indica* inoculation on antioxidant enzymes activity and morphological characteristics of green beans (*Phaseoluse vulgaris* L.) in chilling stress (In Persian). J. Plant Process Funct..

[26] Jiang W., Pan R., Wu C., Xu L., Abdelaziz M.E., Oelmüller R., Zhang W. (2020). *Piriformospora indica* enhances freezing tolerance and post-thaw recovery in *Arabidopsis* by stimulating the expression of *CBF* genes. Plant Signal. Behav..

[27] David B.M., Sun X., Mensah R.A., Li D., Liu F., Tian N., Lai Z., Cheng C. (2020). Physiological and biochemical mechanism of *Piriformospora indica* induced high temperature resistance in banana. Chinese J. Appl. Environ. Biol..

[28] Madaan G., Gosal S.K., Gosal S.S., Saroa G.S., Gill M.I.S. (2013). Effect of microbial inoculants on the growth and yield of micropropagated banana (*Musa indica*) cv. Grand Naine. J. Hortic. Sci. Biotechnol..

[29] Li D., Mensah R.A., Liu F., Tian N., Qi Q., Yeh K.W., Xuhan X., Cheng C., Lai Z. (2019). Effects of *Piriformospora indica* on rooting and growth of tissue-cultured banana (*Musa acuminata* cv. Tianbaojiao) seedlings. Sci. Hortic..

[30] Cheng C., Li D., Qi Q., Sun X., Mensah R.A., David B.M., Zhang Y., Hao X., Zhang Z., Lai Z. (2020). The root endophytic fungus *Serendipita indica* improves resistance of banana to *Fusarium oxysporum* f. sp. *cubense* tropical race 4. Eur. J. Plant Pathol..

[31] Hui F., Peng B., Lou B., Lin F., Nie C., Liu J. (2014). *Piriformospora indica* improves salt tolerance in *Nicotiana tobacum* by promoting the synthesis of osmolyte and inducing the expression of stress resistance genes. J. Agric. Biotechnol..

[32] Sun C., Johnson J.M., Cai D., Sherameti I., Oelmüller R., Lou B. (2010). *Piriformospora indica* confers drought tolerance in Chinese cabbage leaves by stimulating antioxidant enzymes, the expression of drought-related genes and the plastid-localized CAS protein. J. Plant Physiol..

[33] Xu L., Wang A., Wang J., Wei Q., Zhang W. (2017). *Piriformospora indica* confers drought tolerance on *Zea mays* L. through enhancement of antioxidant activity and expression of drought-related genes. Crop J..

[34] Tsai H.J., Shao K.H., Chan M.T., Cheng C.P., Yeh K.W., Oelmüller R., Wang S.J. (2020). *Piriformospora indica* symbiosis improves water stress tolerance of rice through regulating stomata behavior and ROS scavenging systems. Plant Signal. Behav..

[35] Feng X., Lai Z., Lin Y., Lai G., Lian C. (2015). Genome-wide identification and characterization of the superoxide dismutase gene family in *Musa acuminata* cv. Tianbaojiao (AAA group). BMC Genom..

[36] Zhang M. (2010). Cloning and Expression Analysis of Cold Resistance Relative Genes of the Wild Banana (*Musa* spp., AB Group). Ph.D. Thesis.

[37] Yang Y. (2014). Cloning and Expression of *WHIRLY* in the Wild Banana from Sanming City and *Musa* spp. cv. Tianbaojiao. Master’s Thesis.

[38] Liu W. (2018). Studies on the Responsive Mechanism of Wild Banana (*Musa itinerans*) to Low-Temperature Stress Based on the Transcription of RNA-seq Technology. Ph.D. Thesis.

[39] Liu W., Cheng C., Chen F., Ni S., Lin Y., Lai Z. (2018). High-throughput sequencing of small RNAs revealed the diversified cold-responsive pathways during cold stress in the wild banana (*Musa itinerans*). BMC Plant Biol..

[40] Liu W., Cheng C., Lai G., Lin Y., Lai Z. (2015). Molecular cloning and expression analysis of *KIN10* and cold-acclimation related genes in wild banana ‘Huanxi’ (*Musa itinerans*). Springerplus.

[41] Liu W., Lin Y., Lai Z. (2017). Cloning and expression profle of the cDNA and promoter of *CBF7* in wild banana (*Musa itinerans*) from Huanxi of Fuzhou City. Chin. J. Appl. Environ. Biol..

[42] Wu J., Zhang Y., Yin L., Qu J., Lu J. (2014). Linkage of cold acclimation and disease resistance through plant–pathogen interaction pathway in *Vitis amurensis* grapevine. Funct. Integr. Genom..

[43] Chen F. (2016). Cloning and Cold Resistance Analysis of β-1,3 Glucanase Gene (*Mugsps*) from Wild Banana. Master’s Thesis.

[44] Ehlert B., Hincha D.K. (2008). Chlorophyll fluorescence imaging accurately quantifies freezing damage and cold acclimation responses in *Arabidopsis* leaves. Plant Methods.

[45] Chen X., Song F., Zhu X., Sun L., Ma F., Liu S. (2014). Effect of arbuscular mycorrhizal fungus on morphology, growth and photosynthetic characteristics in maize seedlings under low temperature stress. Acta Agric. Boreali Sin..

[46] Cao Y., Dai P., Dai S. (2016). Effects of arbuscular mycorrhiza fungi on seedlings growth and chlorophyll fluorescence parameters in cucumber under low temperature stress. J. Hebei Agric. Sci..

[47] Johnson J.M., Alex T., Oelmüller R. (2014). *Piriformospora indica*: The versatile and multifunctional root endophytic fungus for enhanced yield and tolerance to biotic and abiotic stress in crop plants. J. Trop. Agric..

[48] Johnson J.M. (2014). The Role of Cytosolic Calcium Signaling in Beneficial and Pathogenic Interactions in *Arabidopsis thaliana*. Ph.D. Thesis.

[49] Liu Z., Cheng R., Xiao W., Guo Q., Wang N. (2014). Effect of off-season flooding on growth, photosynthesis, carbohydrate partitioning, and nutrient uptake in *Distylium chinense*. PLoS ONE.

[50] Nath M., Bhatt D., Prasad R., Gill S.S., Anjum N.A., Tuteja N. (2016). Reactive oxygen species generation-scavenging and signaling during plant-arbuscular mycorrhizal and *Piriformospora indica* interaction under stress condition. Front. Plant Sci..

[51] Garg N., Aggarwal N. (2012). Effect of mycorrhizal inoculations on heavy metal uptake and stress alleviation of *Cajanus cajan* (L.) Millsp. genotypes grown in cadmium and lead contaminated soils. Plant Growth Regul..

[52] Khazaei M., Maali-Amiri R., Talei A.R., Ramezanpour S. (2015). Differential transcript accumulation of dhydrin and beta-glucosidase genes to cold-induced oxidative stress in chickpea. J. Agric. Sci. Technol..

[53] Han B., He C., Yan Y., Guo S., Yu X. (2011). Effects of arbuscular mycorrhiza fungi on seedlings growth and antioxidant systems of leaves in cucumber under low temperature stress. Sci. Agric. Sin..

[54] Liu X.M., Xu Q.L., Li Q.Q., Zhang H., Xiao J.X. (2017). Physiological responses of the two blueberry cultivars to inoculation with an arbuscular mycorrhizal fungus under low-temperature stress. J. Plant Nutr..

[55] May M.J., Vernoux T., Leaver C., Montagu M.V., Inze D. (1998). Glutathione homeostasis in plants: Implications for environmental sensing and plant development. J. Exp. Bot..

[56] Sharma P., Kharkwal A.C., Abdin M.Z., Varma A. (2017). *Piriformospora indica* -mediated salinity tolerance in *Aloe vera* plantlets. Symbiosis.

[57] Alscher R.G. (2002). Role of superoxide dismutases (SODs) in controlling oxidative stress in plants. J. Exp. Bot..

[58] Kumar M., Sharma R., Jogawat A., Singh P., Dua M., Gill S.S., Trivedi D.K., Tuteja N., Verma A.K., Oelmüller R. (2012). Piriformospora indica, a root endophytic fungus, enhances abiotic stress tolerance of the host plant. Improving Crop Resistance to Abiotic Stress.

[59] Gill S.S., Gill R., Trivedi D.K., Anjum N.A., Sharma K.K., Ansari M.W., Ansari A.A., Johri A.K., Prasad R., Pereira E. (2016). *Piriformospora indica*: Potential and significance in plant stress tolerance. Front. Microbiol..

[60] Rai M., Acharya D., Singh A., Varma A. (2001). Positive growth responses of the medicinal plants *Spilanthes calva* and *Withania somnifera* to inoculation by *Piriformospora indica* in a field trial. Mycorrhiza.

[61] Yang Q.S., Gao J., He W.D., Dou T.X., Ding L.J., Wu J.H., Li C.Y., Peng X.X., Zhang S., Yi G.J. (2015). Comparative transcriptomics analysis reveals difference of key gene expression between banana and plantain in response to cold stress. BMC Genom..

[62] Sun Y., Li M., Mitra S., Muhammad R.H., Debnath B., Lu X., Jian H., Qiu D. (2018). Comparative phytochemical profiles and antioxidant enzyme activity analyses of the southern highbush blueberry (*Vaccinium corymbosum*) at different developmental stages. Molecules.

[63] Lin Y.L., Lai Z.X. (2013). Superoxide dismutase multigene family in longan somatic embryos: A comparison of *CuZn-SOD*, *Fe-SOD*, and *Mn-SOD* gene structure, splicing, phylogeny, and expression. Mol. Breed..

[64] Chen L., Zhong H.Y., Kuang J.F., Li J.G., Lu W.J., Chen J. (2011). Validation of reference genes for RT-qPCR studies of gene expression in banana fruit under different experimental conditions. Planta.

